# Lumbopelvic stabilization-based physiotherapy and rehabilitation and urotherapy for lower urinary tract dysfunction in Duchenne Muscular Dystrophy: a randomized controlled trial

**DOI:** 10.1016/j.jped.2026.101511

**Published:** 2026-02-13

**Authors:** Demet Öztürk, Aynur Ayşe Karaduman, Türkan Akbayrak

**Affiliations:** a Lokman Hekim University, Faculty of Health Sciences, Department of Physiotherapy and Rehabilitation, Ankara, Turkey; b Hacettepe University, Faculty of Physical Therapy and Rehabilitation, Ankara, Turkey

**Keywords:** Duchenne muscular dystrophy, Lower urinary tract symptoms, Urotherapy, Physical Therapy Modalities

## Abstract

**Objective:**

This randomized controlled trial investigated the effectiveness of supervised lumbopelvic stabilization and urotherapy on factors associated with lower urinary tract symptoms in children with Duchenne Muscular Dystrophy and lower urinary tract dysfunction (LUTD).

**Method:**

The study included 32 children aged 5–12 years, who were ambulatory and had a Dysfunctional Voiding and Incontinence Symptom Scale (DVISS) of 8.5 or higher. Lower urinary tract symptoms were assessed using the DVISS, a three-day bladder diary, and a nocturnal enuresis diary. Bowel symptoms were assessed with a seven-day bowel diary. Moreover, physical performance tests, the strength of pelvis-related muscle groups, and participation in activities of daily living were evaluated using validated measurement tools. Participants were randomized into two groups: the active control group (n:16, weight:25.81±9.53 kg, height:121.25±18.22 cm) received only urotherapy, while the treatment group (n:16, weight:25.88±9.67 kg, height: 122.69±16.63 cm) additionally performed lumbopelvic stabilization-based exercises for eight weeks under the supervision of a physiotherapist.

**Results:**

Urotherapy significantly reduced LUTD severity (*p* < 0.001), enuresis frequency (*p* = 0.014), and the number of symptoms associated with LUTD (*p* = 0.001). However, adding lumbopelvic stabilization exercises conferred no superiority over urotherapy alone (*p* > 0.05). Supervised lumbopelvic stabilization improved functional mobility (*p* < 0.003), muscle strength (*p* < 0.05), and participation in activities of daily living (*p* < 0.049). However, neither urotherapy nor lumbopelvic stabilization training had a significant effect on Gower’s time or daytime voiding frequency (*p* > 0.05).

**Conclusion:**

Both interventions were effective in improving bladder and bowel outcomes in children with Duchenne Muscular Dystrophy and LUTD, with no superiority observed on lower urinary tract symptoms.

## Introduction

Duchenne muscular dystrophy (DMD) is the most common inherited neuromuscular disorder, affecting approximately one in 3500–5000 live male births [1]. It is an X-linked recessive and ultimately fatal disease caused by mutations in the DMD gene, which encodes dystrophin [2]. Dystrophin is a structural protein essential for muscle fiber stability, linking actin microfilaments to the extracellular matrix and stabilizing the sarcolemma during muscle contraction and relaxation [3]. Because dystrophin is expressed in striated, smooth, and cardiac muscle, its deficiency primarily affects the musculoskeletal and cardiovascular systems [4].

Until recently, treatment options for individuals with DMD were largely limited to corticosteroid therapy and cardiopulmonary support. However, advances in medical genetics, improvements in quality of care, expanded rehabilitation opportunities, and technological developments have significantly increased life expectancy in this population [4]. Individuals with DMD, who were previously expected to die before adulthood, are now living into their fourth decade [5,6]. As a result, health problems that were previously overlooked due to the shorter life span of individuals with DMD have gained increasing importance, including bladder and bowel health [7]. Although dystrophin deficiency primarily affects striated muscle, it can also impair smooth muscle function [8]. Consequently, destructive effects on the detrusor muscle and urethral smooth muscle contribute to the high prevalence of lower urinary tract symptoms (LUTS) and lower urinary tract dysfunction (LUTD) in children with DMD [9]. Disease-related factors such as decreased muscle strength during functional movements and reduced functional levels of the upper and lower extremities have also been associated with an increased incidence of LUTS and LUTD. Recent studies have reported that approximately 85–87 % of children with DMD experience at least one LUTS, while nearly 44 % are present with LUTD, highlighting the substantial burden of bladder and bowel dysfunction in this population [7,10].

Urotherapy is the first-line treatment for children with LUTS. It consists of non-invasive interventions and does not involve surgical or pharmacological treatments, making it suitable for use in primary care settings [11]. Lumbopelvic stabilization exercise training aims to improve strength in the lumbopelvic musculature, maintain postural control, enhance pelvic floor muscle function, and reduce urinary symptoms through coordinated muscle co-contraction [12]. No studies have been found in the literature on the use of urotherapy and lumbopelvic stabilization exercises in individuals with DMD. Furthermore, the number of studies on treatment for individuals with DMD and LUTD is insufficient [7,9]. Therefore, this study aimed to examine the effectiveness of supervised lumbopelvic stabilization, which addresses factors associated with LUTS in children with DMD and LUTD.

## Methods

The primary outcome of the study was the severity of LUTD, assessed using the DVISS, to evaluate the effect of lumbopelvic stabilization exercises combined with urotherapy. Secondary outcomes included the effects of a supervised lumbopelvic stabilization exercise program, in addition to urotherapy, on lower urinary tract symptoms, bowel symptoms, physical performance, muscle strength, and participation in activities of daily living.

### Design

This study was designed as a prospective, single-blind, parallel-group, and randomized controlled trial. It aimed to investigate the effects of urotherapy and a lumbopelvic stabilization-based physiotherapy and rehabilitation program on LUTS in children with DMD and LUTD. The study objectives were formulated according to the PICOS framework, as follows:

*P(Population):* Ambulatory children aged ≥ 5 years with DMD and LUTD.

*I(Intervention):* Urotherapy and supervised lumbopelvic stabilization-based exercise program combined with urotherapy.

*C(Comparison):* Comparison of two treatment approaches to evaluate their relative effectiveness.

*O(Outcome):* Changes in LUTS following the interventions.

*S(Study type):* Randomized controlled trial.

The study was approved by the Scientific Research Ethics Committee of Lokman Hekim University (October 31, 2024; approval no 232) and registered at ClinicalTrials.gov (NCT06643923). The trial was conducted in accordance with the Declaration of Helsinki. Written informed consent for participation and publication was obtained from all participants and their caregivers. The study was reported in compliance with the CONSORT guidelines [13].

### Participants

**Fig. 1:**
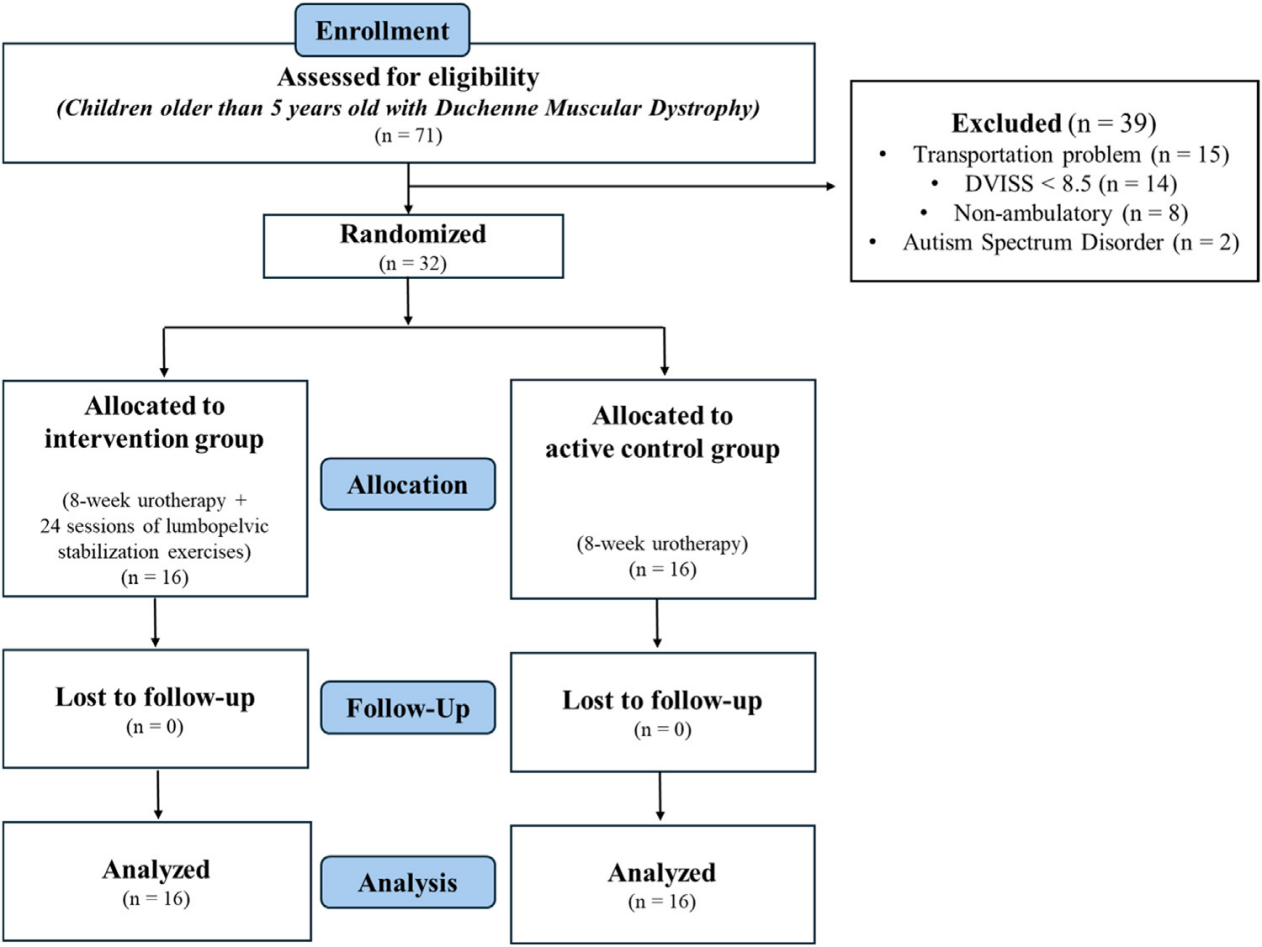
CONSORT flow diagram of the study.

The study included ambulatory children aged > 5 years with a diagnosis of DMD who were recruited between November 2024 and July 2025. Children with DMD who scored ≥ 8.5 on the Dysfunctional Voiding and Incontinence Symptom Scale (DVISS) and were classified within the first four stages of the Vignos Scale were eligible for inclusion. Exclusion criteria included the presence of comorbid conditions (e.g., Becker muscular dystrophy or autism spectrum disorder), cooperation difficulties, and lack of adequate internet infrastructure. Of the 71 patients admitted to the Muscular and Nerve Diseases Application and Research Center in XXX University, 39 were excluded. The treatment and active control groups were 16 and 16, respectively. Consequently, the study was completed with 32 children diagnosed with DMD and their families (Figure 1).

Sample size estimation was based on right hip flexor strength data reported by Kenis-Coskun et al. (2022). Assuming 95 % power and a two-sided α of 0.05, a minimum of 15 participants per group was required [14]. A post-hoc power analysis for the ANCOVA of dominant hip flexor strength yielded an observed power of 81.17 % (effect size *F* = 0.520, α = 0.05, total sample size = 32).

### Outcome measures

Participants’ demographic and disease-related characteristics were collected using a general assessment form developed by the researchers. All assessments were conducted by the same experienced physiotherapist, under identical conditions, both before and after the interventions. Accordingly, due to the nature of the study, the physiotherapist who performed the baseline and final assessments could not be blinded.

The DVISS questionnaire was employed both as an inclusion criterion and as an outcome measure. This questionnaire consists of 13 items evaluating 11 LUTS, focusing on the presence and severity of symptoms within the past month. The inclusion criterion of the DVISS score ≥ 8.5 was based on the original validation study by Akbal et al., which demonstrated that a score of 8.5 or higher represents the optimal cut-off for diagnosing LUTD in male children. Accordingly, this validated threshold was used to identify clinically meaningful LUTD, and children with DMD scoring ≥ 8.5 on the DVISS were invited to participate in the study [15]. Moreover, a three-day bladder diary and a nocturnal enuresis diary were also utilized [16,17].

Bowel symptoms were assessed using a seven-day bowel diary. The diary recorded data on defecation frequency, timing and pain intensity during defecation [16]. Both the bladder diary and the bowel diary were completed by the family, based on direct observation of the child as well as information obtained from the child.

Physical performance was assessed using the Timed Up and Go (TUG) test and the Gowers’ test, both validated for individuals with DMD [18]. The TUG test measured the time required to stand from a chair, walk 3 m, turn, and sit down, while the Gowers’ test assessed the time needed to rise from a supine position to standing [19]. Each test was performed twice, and the best score was recorded to minimize learning effects.

Muscle strength of trunk flexors, trunk extensors, hip flexors, and knee extensors were assessed using the microFET2 handheld dynamometer (Hoggan Scientific, LLC, Salt Lake City, UT, USA). The make test was applied at standardized starting positions, with participants performing a four-second maximal contraction. Three trials were completed for each muscle group, and mean values were recorded in kilograms. Testing positions were chosen based on established reliability [9,20].

Activities of daily living (ADL) participation and independence were evaluated using the 10-item Barthel Index, with scores ranging from 0 to 100 and higher scores indicating greater independence [21].

### Randomization

Block randomization with a fixed block size of six and a 1:1 allocation ratio was used to ensure balanced group sizes throughout participant enrollment. An independent researcher generated the randomization lists via a randomization program (https://www.sealedenvelope.com/simple-randomiser/v1/lists). Lists were placed in sealed, opaque envelopes and provided to the principal investigator, who opened them sequentially as participants were enrolled, assigning each to either the treatment or active control group. The researcher assigning the groups did not have access to the random allocation sequence. Due to the nature of the study, participants and the implementing researcher could not be blinded. To ensure study quality and reduce bias, the statistician conducting the analyses was blinded. Moreover, to minimize potential performance and contamination bias, all participants were recruited and treated by the same physiotherapist using predefined and standardized protocols. Appointments were scheduled to prevent interaction between groups, and both interventions were presented as standard and potentially beneficial approaches.

### Intervention

Before initiating the treatment programs, both groups received an individualized, face-to-face urotherapy session lasting approximately 90 min, tailored to each child’s symptoms. The topic headings addressed in the urotherapy education are summarized in Table 1. The recommendations of the ICCS guidelines were followed during all stages of the urotherapy. All participants were asked to implement the lifestyle modifications learned during this training over the course of eight weeks [11].

**Table 1:** Content of the urotherapy education program.

Urotherapy Topics	
Urinary system anatomy	Regulation of voiding frequency
Urinary system physiology	Teaching optimal voiding posture
Bowel system physiology	Teaching optimal defecation posture
Pelvic floor anatomy, physiology, and awareness	Behavioral recommendations for bowel health
Definitions of lower urinary tract symptoms	Proper straining techniques
Etiology of lower urinary tract symptoms	Nutritional recommendations (bladder irritants, fiber intake, fluid intake)
Association between lower urinary tract symptoms and Duchenne	Recommendations for communication with the child
Importance of child and family education	Strategies to increase physical activity
Regulation of water intake	Genital hygiene
Teaching abdominal massage	Benefits of breathing exercises
Benefits of trunk exercises	Benefits of stretching exercises
Awareness of nocturnal alarm therapy	Mindfulness and meditation techniques

Regular recording and frequent communication with the physiotherapist are essential for effective urotherapy. Therefore, four strategies were employed to enhance adherence and motivation: signing a compliance contract, a star-reward system, balloon-coloring tracking, and reminder messages [11].

**Fig. 2:**
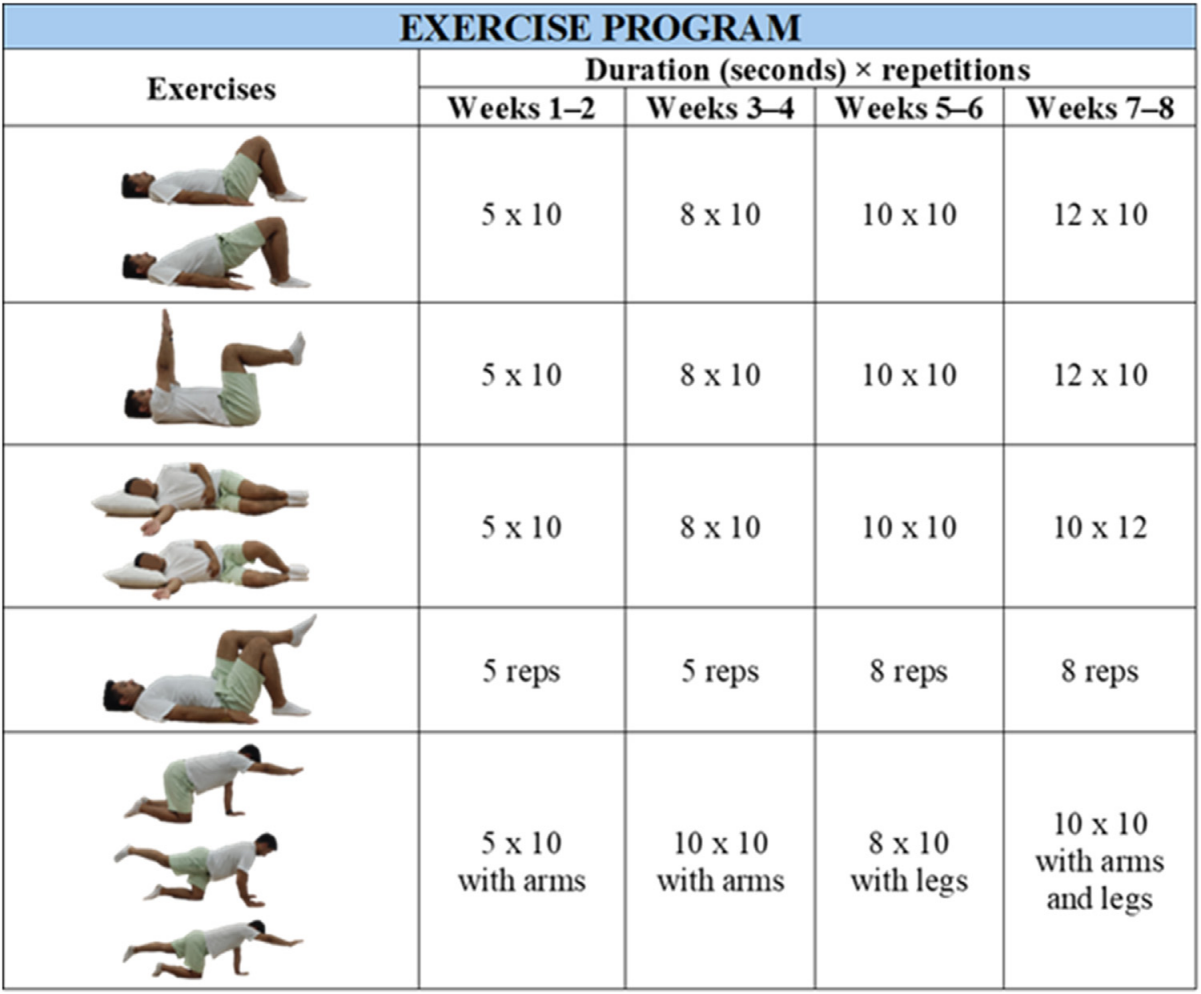
Lumbopelvic stabilization-based exercise program of intervention group.

The active control group received only urotherapy training, which is considered the gold standard in the management of pediatric LUTS [11]. The treatment group, in addition to urotherapy, received a lumbopelvic stabilization exercise program. All lumbopelvic stabilization exercises were delivered synchronously via online video conferencing platforms under the real-time supervision of an experienced physiotherapist. Exercise performance was continuously monitored, and incorrect movements were immediately corrected to ensure proper technique and intervention fidelity. The exercise program was designed according to the FITTEA principle and was progressive (Figure 2) [22]. Sessions were conducted three times per week, each lasting approximately 30 min (5 min of warm-up, 20 min of exercise, and 5 min of cool-down; in a way that does not cause fatigue), for a total duration of eight weeks. Consequently, the treatment group performed 24 supervised trunk stabilization exercise sessions in addition to adhering to their urotherapy tasks.

### Statistical analysis

Statistical analyses were performed using IBM SPSS Statistics version 26.0. Continuous variables were analyzed using independent or paired *t*-tests, or Mann–Whitney U and Wilcoxon signed-rank tests, as appropriate. Categorical variables were analyzed using chi-square, Fisher’s exact, or McNemar tests. Effect sizes were calculated using Cohen’s d for parametric tests and Z/√n for non-parametric tests. Between-group intervention effects were assessed using one-way ANCOVA with baseline values as covariates; Quade’s ANCOVA was applied when assumptions were violated. Effect sizes were interpreted using partial eta squared (η²). A two-sided p-value < 0.05 was considered statistically significant. No missing data was observed.

## Results

Thirty-two children with DMD, aged 5–12 years, Vignos stages 1–4, and with a DVISS ≥ 8.5, were enrolled. The treatment (*n* = 16) and active control groups (*n* = 16) were comparable in demographic and clinical characteristics, including age, anthropometrics, DVISS scores, physiotherapy frequency, Vignos stage, corticosteroid use, scoliosis, and toilet habits (*p* > 0.05) (Table 2).

**Table 2:** Sociodemographic and clinical characteristics of the participants.

	**Intervention Group (*n* = 16)**	**Active Control Group (*n* = 16)**	**p**
**Age** (years)	8.94 ± 2.24	8.12 ± 2.50	0.340
**Body weight** (kg)	25.88 ± 9.67	25.81 ± 9.53	0.985
**Height** (cm)	122.69 ± 16.63	121.25 ± 18.22	0.817
**BMI** (kg/m^2^)	15.81 (11.87 / 26.97)	16.65 (12.84 / 22.05)	0.438
**DVISS total score**	13.50 (9/25)	11.50 (9/22)	0.383
**Toilet training age** (years)	2.00 (1 / 4)	2.75 (1 / 5)	0.098
**Number of physiotherapy sessions received** (times/week)	2 (0 / 4)	2 (0 / 4)	0.548
**Vignos Scale**			0.478
Stage 1	6 (37.50)	7 (43.75)	
Stage 2	4 (25.00)	6 (37.50)	
Stage 3	6 (37.50)	3 (18.75)	
Stage 4	0 (0.00)	0 (0.00)	
**Using steroid**			0.101
Yes	16 (100)	12 (75)	
No	0 (0)	4 (25)	
Quit	0 (0)	0 (0)	
**Scoliosis**			NC
Yes	0 (0)	0 (0)	
No	16 (100)	16 (100)	
**Toilet habits**			NC
Toilet	16 (100)	16 (100)	
Other (diapers, catheters, etc.)	0 (0)	0 (0)	

Data are presented as mean ± SD, median (minimum/maximum), or n ( %). x̄, Mean, SD, Standard deviation, n, Frequency, %, Percentage, BMI, Body Mass Index, DVISS, Dysfunctional Voiding and Incontinence Symptom Scale, NC, Not calculable, *p*, Independent Samples *t*-test, Mann–Whitney U test, or Chi-square test.

Compared with baseline, both the treatment and active control groups showed significant reductions in DVISS total scores and enuresis frequency (*p* < 0.05). A significant decrease in TUG time was observed only in the treatment group (*p* < 0.05), whereas no significant changes were detected in Gower’s test duration in either group (*p* > 0.05). Between-group comparisons revealed no significant differences in DVISS total scores, enuresis frequency, or Gower’s test duration (*p* > 0.05). However, the treatment group demonstrated a significantly greater improvement in TUG time, indicating a benefit in physical performance outcomes (Table 3).

**Table 3:** Descriptive statistics and within- and between-group comparisons in terms of DVISS total score, enuresis frequency, and physical performance parameters.

Parameter	Statistics					
DVISS total score	Descriptive data					
	**Intervention Group** **(*n* = 16)**			**Control Group** **(*n* = 16)**		
	**Pre**	**Post**		**Pre**		**Post**
	13.50 (9/25)	3.00 (1/13)		11.50 (9/22)		4.00 (0/14)
	**Within-group changes**					
	**Intervention Group** **(*n* = 16)**			**Control Group** **(*n* = 16)**		
	p*	**ES**		p*		**ES**
	**<0.001**	0.643		**0.001**		0.627
	**Comparison of changes between groups**					
	**F_1.29_**		p**		**ES**	
	0.887		0.354		0.029	

**Enuresis frequency**	**Descriptive data**					
	**Intervention Group** **(*n* = 16)**			**Control Group** **(*n* = 16)**		
	**Pre**	**Post**		**Pre**		**Post**
	1.00 (0/7)	0.00 (0/7)		0.50 (0/3)		0.00 (0/3)
	**Within-group changes**					
	**Intervention Group** **(*n* = 16)**			**Control Group** **(*n* = 16)**		
	p*	**ES**		p*		**ES**
	**0.033**	0.389		**0.014**		0.449
	**Comparison of changes between groups**					
	**F_1.29_**		p**		**ES**	
	0.006		0.941		0.000	

**TUG time** (*sec*)	**Descriptive data**					
	**Intervention Group** **(*n* = 16)**			**Control Group** **(*n* = 16)**		
	**Pre**	**Post**		**Pre**		**Post**
	8.31 (6.16 / 20.61)	7.60 (5.05 / 18.14)		6.70 (4.77 / 18.55)		6.68 (5.01 / 22.26)
	**Within-group changes**					
	**Intervention Group** **(*n* = 16)**			**Control Group** **(*n* = 16)**		
	p*	**ES**		p*		**ES**
	**0.003**	0.538		0.535		0.113
	**Comparison of changes between groups**					
	**F_1.29_**		p**		**ES**	
	9.368		**0.005**		0.238	

**Gower’s time** (*sec*)	**Descriptive data**					
	**Intervention Group** **(*n* = 16)**			**Control Group** **(*n* = 16)**		
	**Pre**	**Post**		**Pre**		**Post**
	5.50 (2.32 / 43.68)	5.40 (2.34 / 40.51)		4.39 (1.75 / 52)		4.97 (1.84 / 50)
	**Within-group changes**					
	**Intervention Group** **(*n* = 16)**			**Control Group** **(*n* = 16)**		
	p*	**ES**		p*		**ES**
	0.053	0.353		0.196		0.236
	**Comparison of changes between groups**					
	**F_1.29_**		p**		**ES**	
	1.144		0.294		0.038	

Values are presented as median (minimum/maximum). p*, The Wilcoxon Signed-Rank Test, p**, Quade’s ANCOVA. DVISS, Dysfunctional Voiding and Incontinence Symptom Scale; TUG, Time Up and Go; *sec*, Seconds; Pre, Before treatment; Post, After treatment; ES, Effect size.

Within-group analyses demonstrated significant improvements in the treatment group for trunk flexor (*p* < 0.001) and extensor strength (*p* = 0.006), dominant (*p* = 0.010) and non-dominant (*p* = 0.011) quadriceps femoris strength, as well as dominant and non-dominant hip flexor strength (both *p* < 0.001). In the active control group, a significant improvement was observed only in non-dominant quadriceps femoris strength (*p* = 0.046). Both groups showed improvements in Barthel Index scores. Between-group comparisons revealed no significant differences in changes in trunk flexor strength, trunk extensor strength, or non-dominant knee extensor strength (*p* > 0.05). However, improvements in dominant quadriceps femoris (*p* = 0.036), dominant hip flexor (*p* = 0.009), non-dominant hip flexor strength (*p* = 0.031), and Barthel Index total scores were significantly greater in the treatment group (Table 4).

**Table 4:** Intragroup comparison of pre- and post-treatment muscle strengths and Barthel Index total score.

	**Intervention Group (*n* = 16)**				**Active Control Group (*n* = 16)**				ANCOVA*		
	**Pre**	**Post**	**p**	**ES**	**Pre**	**Post**	**p**	**ES**	**F_1,29_**	**p**	**ES**
**Trunk flexor MS**	5.73 ± 1.33	7.05 ± 1.34	**<0.001**	1.277	5.66 ± 2.33	5.80 ± 2.03	0.411	0.211	3.139	0.087	0.098
**Trunk extensor MS**	5.99 ± 1.86	6.68 ± 1.70	**0.006**	0.795	5.59 ± 1.75	5.74 ± 2.29	0.593	0.136	2.434	0.130	0.077
**Quadriceps femoris MS (D)**	3.72 ± 1.95	4.75 ± 2.49	**0.010**	0.739	4.92 ± 2.75	5.11 ± 3.39	0.527	0.162	4.830	**0.036**	0.143
**Quadriceps femoris MS (ND)**	3.94 ± 2.11	4.85 ± 2.54	**0.011**	0.724	4.32 ± 2.19	4.81 ± 2.73	**0.046**	0.543	1.462	0.236	0.048
**Hip flexor MS (D)**	3.90 ± 1.12	5.22 ± 1.35	**<0.001**	1.271	4.26 ± 1.98	4.53 ± 2.06	0.302	0.267	7.847	**0.009**	0.213
**Hip flexor MS (ND)**	3.21 ± 0.87	4.13 ± 0.76	**<0.001**	1.349	3.63 ± 1.40	3.86 ± 1.72	0.341	0.245	5.152	**0.031**	0.151
**Barthel Index total score**	82.19 ± 7.06	89.06 ± 6.64	**<0.001**	1.436	75.31 ± 18.93	78.75 ± 19.02	**0.029**	0.603	4.209	**0.049**	0.127

Data are presented as mean ± SD. x̄, mean; SD, standard deviation; *p*, Paired *t*-test, used to compare within-group pre- and post-treatment values. D, Dominant; ND, Non-dominant; Pre, Before treatment; Post, After treatment; ES, Effect size. * Data are presented as mean ± SE. x̄, mean; SE, standard error. The p-value of ANCOVA was used to compare the effectiveness of the treatments. Post-treatment values were assigned as the dependent variable, the grouping variable as the independent variable, and pre-treatment values as the covariate. Muscle strength values are presented in kilograms. MS, Muscle strength; D, Dominant; ND, Non-dominant.

Analysis of bladder diary data (urination frequency, number of lower urinary tract symptoms, and daily fluid intake) and bowel diary data (defecation frequency and pain intensity during defecation) revealed a statistically significant reduction in the number of symptoms in both groups (*p* < 0.05). Between-group comparisons showed no significant differences in these variables, except for defecation frequency (*p* > 0.05) (Table 5).

**Table 5:** Intra- and inter-group comparison of bladder diary and bowel diary data.

	Intervention Group (*n* = 16)				Active Control Group (*n* = 16)				Quade’s ANCOVA		
	Pre	Post	p	ES	Pre	Post	p	ES	F_1,29_	p	ES
**Urination frequency** (number)	5 (3/8)	5 (4/7)	0.475	0.130	4 (2/9)	5.5 (4/7)	0.502	0.123	0.137	0.714	0.005
**LUTS** (number)	8 (4/14)	3 (1/5)	**<0.001**	0.645	8 (4/10)	3.5 (1/7)	**0.001**	0.624	0.237	0.630	0.008
**Daily fluid intake** (liters)	766.5 (466 / 1600)	950 (400 / 1900)	0.098	0.302	450 (100 / 3000)	700 (200 / 3000)	**0.005**	0.516	0.118	0.734	0.004
**Defecation frequency** (number)	6 (2/7)	6 (3/7)	0.101	0.300	6 (3/7)	7 (6/7)	**0.011**	0.463	11.572	**0.002**	0.278
**Pain intensity during defecation**	0 (0/9)	0 (0/0)	0.068	0.333	0 (0/6)	0 (0/4)	**0.034**	0.387	1.505	0.229	0.048

Values are presented as median (minimum/maximum). *p*, The Wilcoxon Signed-Rank Test was used to assess the significance of within-group changes. The p-value of Quade’s ANCOVA was used to compare the effectiveness of the treatments. Post-treatment values were assigned as the dependent variable, the grouping variable as the independent variable, and pre-treatment values as the covariate. LUTS, Lower urinary tract symptoms; Pre, Before treatment; Post, After treatment; ES, Effect size.

## Discussion

In this study investigating the effects of a lumbopelvic stabilization-based exercise program combined with urotherapy in children with DMD and LUTD, it was found that urotherapy alone effectively reduced LUTS severity, enuresis frequency, and the number of associated symptoms. Both urotherapy and the combined exercise intervention were effective for these parameters; however, no additional benefits of combining lumbopelvic stabilization exercises were observed. Neither intervention affected Gower’s time nor daytime voiding frequency. In the urotherapy-only group, daily fluid intake and weekly defecation frequency increased, and defecation-related pain decreased.

The supervised lumbopelvic stabilization program led to improvements in functional movements and increased strength in pelvis-related muscle groups, including trunk flexors/extensors, hip flexors, and knee extensors. Incorporating these exercises into rehabilitation, alongside standard urotherapy, appeared to enhance participation in daily life activities.

To enable physical performance testing and address limitations in previous studies with wide age ranges [7,9], only ambulatory children with DMD aged over five years were included, in line with guideline recommendations [16,23]. Participants’ ages and other demographic or disease-related characteristics were similar across intervention and control groups, enhancing internal validity and homogeneity.

Consistent with the study’s primary hypothesis, both groups showed reductions in LUTD severity, but no significant between-group differences were found, indicating that adding lumbopelvic stabilization exercises did not further reduce LUTS or symptom severity. Previous research supports these findings: remote urotherapy at home has demonstrated significant effects on LUTS in children [24], and urotherapy has been shown to improve uroflowmetry parameters and reduce residual urine volume [25]. Borgström et al. (2022) reported significant reductions in weekly enuresis frequency with standard urotherapy in healthy children [26], and Jørgensen et al. (2023) emphasized the need for controlled studies to clarify urotherapy’s role in pediatric enuresis [27]. Thus, the present findings align with existing literature while providing novel data for the DMD population.

Although lumbopelvic stabilization was expected to improve pelvic floor function and further reduce LUTS, no significant additional effect beyond urotherapy was observed. Nevertheless, in a progressive condition such as Duchenne muscular dystrophy, the absence of symptom deterioration remains clinically meaningful. Preserving urinary function while simultaneously improving physical performance and participation in activities of daily living may therefore represent a relevant therapeutic goal in this population. Moreover, in this study, the authors focused on abdominal stabilizer muscles because of their influence on the pelvic floor. Since the authors anticipated that these children might perform pelvic floor exercises incorrectly, it was not included in the program. Instead, the authors focused on the thoracic diaphragm and transversus abdominis muscles to indirectly influence the pelvic floor, which may explain the observed outcomes. Nevertheless, in a progressive condition such as DMD, the implemented core stabilization exercises at least halted the progression of LUTS and did not exacerbate them, indicating that this intervention can be considered safe for children with DMD and LUTD [5].

Daily fluid intake increased significantly only in the urotherapy group, whereas no significant change was observed in the exercise group. This may be explained by the additional weekly exercise sessions in the latter, adding responsibilities beyond the standard urotherapy tasks. Moreover, although bladder diaries are recommended by guidelines, they capture only the assessed week and may not fully reflect the child’s routine [28]. Therefore, conclusions based solely on bladder diaries should be interpreted with caution.

In the present study, urotherapy in children with DMD and LUTD led to increased weekly defecation frequency and reduced pain during defecation. This effect is likely related to softer stool consistency resulting from lifestyle modifications. Shiro et al. reported a significant association between stool consistency, as assessed by the Bristol Stool Scale, and perceived pain, supporting this interpretation [24]. Moreover, behavior changes such as adequate hydration, avoidance of postponement maneuvers, increased pelvic floor awareness, and higher physical activity levels are expected to positively influence bowel health [29]. These findings are consistent with previous research. Bowel outcomes were assessed as secondary measures due to their close association with lower urinary tract dysfunction in pediatric populations. In children with Duchenne muscular dystrophy, bowel dysfunction may contribute to urinary symptoms; therefore, bowel-related findings should be interpreted as clinically relevant complementary outcomes [16].

Improvement in lumbopelvic stabilization enhances proper pelvis and spine alignment during both static and dynamic activities, increases spinal stability through better intra-abdominal pressure control and motor coordination, and reduces strain during physical activity [30]. Shahvarpour et al. [31] reported that an eight-week lumbar stabilization program positively affects movement patterns and motor control, while Jeong et al. [32] demonstrated that good lumbopelvic control improves dynamic stability during gait. Consequently, increased confidence and reduced fear of falling may enhance movement quality [33]. These findings are consistent with the improvements observed in the Timed Up and Go Test in our intervention group.

Lumbopelvic stabilization exercises can enhance spinal stability, improve postural control, and prevent common spinal deformities in children with DMD, although their importance is not yet emphasized in disease management guidelines [23]. While muscle strength increased in all assessed groups, significant differences between-groups were observed only in the dominant quadriceps femoris and bilateral hip flexors, likely due to approximately 1-kg increases in these muscles [34]. Although such exercises cannot reverse genetic damage or fully eliminate disease symptoms, they help preserve muscle function, increase strength, and delay functional decline [35].

As functional levels progressively decline in DMD, dependence on caregivers increases [36], and the presence of LUTS further impairs participation in ADL [37] Both interventions improved independence, with significantly greater gains in the exercise group. These findings suggest that targeting LUTS is important for enhancing participation in ADL in children with DMD and LUTD.

### Strengths and limitations

This study demonstrates several strengths, including a comprehensive assessment of all lower urinary tract symptoms recommended by ICCS, use of validated and population-appropriate evaluation tools (e.g., Vignos Scale, Barthel Index, hand dynamometry), and selection of a homogeneous participant group with similar functional levels. Moreover, this is the first therapeutic study targeting bladder and bowel health in children with DMD, offering novel contributions to the literature.

Nonetheless, certain limitations exist: long-term outcomes were not studied, only the acute outcome after 8 weeks was evaluated; isolated pelvic floor exercises could not be provided; bladder and bowel diaries may not fully reflect routine behavior; small sample size; and a placebo-controlled design was not feasible, though an active control group and assessor blinding during statistical analysis were implemented to minimize bias. Additionally, non-participation due to practical constraints may have limited the generalizability of the findings. Future studies should address these limitations to evaluate sustained effects and optimize intervention strategies.

Urotherapy alone or combined with a lumbopelvic stabilization-based exercise program effectively reduces bladder- and bowel-related symptoms in children with DMD and LUTD. For greater improvements in physical performance, muscle strength, and participation in ADL, combining lumbopelvic stabilization exercises with urotherapy appears to be the more effective approach.

## Statements relating to our ethics and integrity policies

All procedures performed in studies involving human participants were in accordance with the ethical standards of the institutional research committee and with the 1964 Helsinki declaration and its later amendments or comparable ethical standards. The study was approved by the Scientific Research Ethics Committee of Lokman Hekim University (October 31, 2024; approval no 232) and registered at ClinicalTrials.gov (NCT06643923).

## Data availability statement and data sharing

The entire data set related to the study is available and archived by the authors. With publication, anonymized participant data can be shared upon reasonable request. Requests should be directed by email to the corresponding author (demett.ozturkk@gmail.com). After signing the data access agreement, the corresponding author will share the data of the article.

## Patient consent statement

Each participant was given detailed information about the study, and each participant was asked to sign an informed consent form.

## Funding

This work was supported by the TUBITAK (Project ID: 125S108); and the Lokman Hekim University Scientific Research Projects Coordination Unit (Project ID: THIZ-2025–125).

## CRediT authorship contribution statement

**Demet Öztürk:** Conceptualization, Methodology, Software, Validation, Formal analysis, Investigation, Resources, Data curation, Writing – original draft, Writing – review & editing, Visualization, Project administration, Funding acquisition. **Aynur Ayşe Karaduman:** Methodology, Software, Validation, Investigation, Resources, Data curation, Writing – review & editing. **Türkan Akbayrak:** Conceptualization, Methodology, Software, Validation, Formal analysis, Writing – review & editing, Supervision.

## Conflicts of interest

The authors declare no conflicts of interest.
